# Dissect Gender-Dependent Susceptibility SNPs in Progressive Osteoarthritis Using Regulator Motif Candidate of Genetic Association Strategy (RMCGA)

**DOI:** 10.3390/ijms26094117

**Published:** 2025-04-26

**Authors:** Yin-Shiuan Bai, Ding-Lian Wang, Meng-Chang Lee, Chih-Chien Wang, Wen-Hui Fang, Su-Wen Chuang, Yu-Hsuan Chen, Hao Su, Cheng-Jung Chen, Sui-Lung Su

**Affiliations:** 1 Graduate Institute of Life Sciences, National Defense Medical Center, Taipei 114201, Taiwan; catebai519@gmail.com (Y.-S.B.); dl.wang19971224@gmail.com (D.-L.W.); 2 School of Public Health, National Defense Medical Center, Taipei 114201, Taiwan; apply0710@yahoo.com.tw (M.-C.L.); suwen7@gmail.com (S.-W.C.); jamiechen@mail.ndmctsgh.edu.tw (Y.-H.C.); d131419@gmail.com (H.S.); 3 Department of Orthopedics, Tri-Service General Hospital, National Defense Medical Center, Taipei 114202, Taiwan; tsghcc@gmail.com; 4 School of Medicine, National Defense Medical Center, Taipei 114201, Taiwan; rumaf.fang@gmail.com; 5 Department of Family and Community Medicine, Tri-Service General Hospital, National Defense Medical Center, Taipei 114202, Taiwan; 6 Graduate Institute of Medical Sciences, National Defense Medical Center, Taipei 114201, Taiwan; 7 Division of General Surgery, Department of Surgery, Tri-Service General Hospital, National Defense Medical Center, Taipei 114202, Taiwan; doc20227@yahoo.com.tw; 8 Taichung Veterans General Hospital Chiayi Branch, Chiayi City 60090, Taiwan

**Keywords:** gender-specific, osteoarthritis (OA), NF-κB binding sites, single nucleotide polymorphisms (SNPs)

## Abstract

The role of gender in osteoarthritis (OA) has been reported. However, knowledge on whether gender-specific regulatory SNPs are determining factors in OA is limited. We aimed to identify susceptible gender-specific SNPs of transcription factor binding sites in OA. We used a modified NF-κB binding motif from an RNA sequencing data-inferred OA-associated upstream regulator to define genome-wide potential NF-κB binding sites, which were aligned to the Taiwan BioBank SNP database to identify susceptible SNPs. A case-control study was conducted to verify SNPs with OA determined by a logistic model. The functional assessment was validated using the Genotype-Tissue Expression Portal database. We collected 533 OA patients and 614 healthy controls. Two of nine novel OA-associated SNPs were identified to be significant. For males, the variant of rs73164856 in the aldose reductase gene enhancer was identified to be a protective factor of severe OA patients [odds ratio (OR): 0.17, 95% confidence interval (CI): 0.04–0.73]. For females, the variant of the rs545654 in the neuronal NOS (nNOS) gene was identified to be a detrimental factor of severe OA patients (OR: 2.07, 95% CI: 1.15–3.73). The gene expression analysis demonstrated a lower expression of the AKR1B15 gene (*p* = 0.00019) upon the rs73164856 T allele; meanwhile, it showed a higher expression of the nNOS gene (*p* = 1.2 × 10^−17^) upon the rs545654 T allele. This study identifies susceptible gender-specific SNPs of NF-κB binding sites in severe OA and validates the RMCGA, which sheds light on genetic determinants by gender in advanced OA.

## 1. Introduction

Osteoarthritis (OA) is one of the major causes of disability in the elderly [1]. Recent estimates from the injury and risk factor study by Global Burden of Diseases (GBD) found that the global age-standardized prevalence rate of knee OA was 181.2 per 100,000 [2]. OA is the result of complex interactions between many risk factors [3], including genetic inheritance, diet, obesity, gender, mechanical damage, and age [4], among which there are 45% genetic inheritance for OA [5,6,7]. Findings on gender differences in OA prevalence are inconsistent. Particularly, women are prone to developing OA [8]. Therefore, more research on gender-specific genetic variants is needed for further validation.

The genome-wide association study (GWAS) is a popular approach in disease-related genome research. This method is based on the use of linkage disequilibrium in chromosomes to map disease-causing alleles [9]. However, the association of gene loci by a GWAS remains under debate [10]. An example has shown that a GWAS in osteoarthritis-related studies could only explain approximately 20% of the variation in OA due to missing heritability and stringent statistical test value (*p* < 5 × 10^−8^) [11]. Gallagher et al., 2018, suggested that an increased emphasis on the downstream functional dissection of already-identified GWAS loci, rather than a search for ever more GWAS loci, might be most likely to benefit knowledge of pathophysiology [12].

The pathophysiological causes of OA are complex, with the orchestration of various transcription factors (TFs) and biological pathways forming a complex regulatory network. Studies have identified the NF-κB, an example, as abnormally activated in OA and as a disease-contributing factor [13,14,15,16]. A recent RNA sequencing (RNA-seq) study, the Gene Expression Omnibus (GEO) database, accession number 107,006 (GSE107006), revealed that osteoarthritis-related TF, NF-κB, regulates a pathophysiological network in patients [1]. To our knowledge, polymorphisms in TF binding sites (TFBSs) have been explored for their association with disease [17]. As a result, we established the NF-κB motif based on the core NF-κB motif with some modifications based on Watson–Crick DNA structure rules [18]. To select genome-wide NF-κB binding sites, we used the established NF-κB motif to align the National Center for Biotechnology Information (NCBI) database and to obtain a single nucleotide polymorphism (SNP)-covered NF-κB binding motif by mapping the Taiwan BioBank (TWB) polymorphism database. To further confirm those selective SNPs on theoretical NF-κB binding sites, we used NF-κB chromatin immunoprecipitation sequencing (ChIP-seq) data from the GEO database (GSE55105) to verify and infer candidate SNPs for OA.

To examine whether the Regulator Motif Candidate of Genetic Association Strategy (RMCGA) seeks OA-associated SNPs, we performed a case-control study to investigate the association between the candidate SNPs, particularly gender-oriented variants, and progressive OA. Finally, we used the Genotype-Tissue Expression (GTEx) database to validate the functionality of SNPs.

To investigate gender differences in susceptibility to OA, this study proposes a novel strategy, RMCGA, to identify potentially gender-specific SNPs within TFBSs associated with progressive OA. Through a genetic perspective, we aim to dissect susceptible gender-specific SNPs of transcription factor binding sites in OA.

## 2. Results

### 2.1. Demonstration of Potential NF-κB Binding Motif

The modified NF-κB binding motif, 5′-KGGRMTTYCCM-3′, was shown in Figure S1. Some 33,731 potential binding sites across the whole genome are shown in Figure S2.

### 2.2. Candidate SNPs on NF-κB Binding Sites

According to Fisch et al. [19], an upstream TF, NF-κB, was identified from DEGs associated with OA. We selected NGS data on 1517 samples from the Taiwan BioBank and excluded 13,614,996 SNPs with structural mutations (insertions and deletions) and 14,852,238 SNPs with a sequencing quality control call rate of <90%. As a result, 46,421,352 SNPs were included in this study. The NCBI human genomic sequence hg19 and homologous or motif sequences were used for sequence alignment with the SNPs screened from the Taiwan BioBank. There were 33,731 regions with the NF-κB motif. After excluding the SNPs with a minor allele frequency (MAF) <5%, 919 SNPs remained. ChIP-seq data from the JASPAR database were used to confirm whether the genetic variants had the following combinations of sites: NF-κB (Matrix ID: MA0105.3). Finally, a total of nine SNPs were successfully selected: rs11826681, rs2257609, rs3749606, rs4702701, rs545654, rs7256865, rs73164856, rs77836284, and rs79975923 (Table S1).

### 2.3. Characteristics of OA Patients and Control Subjects

Basic demographics were shown in Table 1. There were 614 healthy individuals in the control group (male = 268, female = 346) and 533 patients in the OA group (male = 195, female = 338). The number of female participants was significantly higher than that of male in the OA group (*p* = 0.015). The age (*p* = 0.043) and body mass index (BMI) (*p* = 0.042) of the OA group were both higher than those of the control group.

### 2.4. Association Between Binding Site Gene Polymorphisms and OA Susceptibility

To highlight the main findings of this study, two gender-specific SNPs showed significant associations with severe OA and gene expression regulation. For males, the variant of rs73164856 in the aldose reductase gene enhancer was identified to be a protective factor of severe OA patients [odds ratio (OR): 0.17, 95% confidence interval (CI): 0.04–0.73]. For females, the variant of the rs545654 in the neuronal NOS (nNOS) gene was identified to be a detrimental factor of severe OA patients (OR: 2.07, 95% CI: 1.15–3.73).

In total, nine SNPs were included in this study. All loci conformed to Hardy–Weinberg equilibrium (*p* > 0.05). In a case-control study of OA, only SNP rs73164856 was significantly protective in OA (Adj-OR: 0.68, 95% CI: 0.50–0.93) (Table 2). However, after stratifying by gender, SNP rs73164856 was found to be significantly protective in males only (Adj-OR: 0.55, 95% CI: 0.33–0.92) (Table S2). In a case-control study of severe OA (KL ≥ 3), rs545654 and rs79975923 showed a significant risk to OA with OR: 1.86 (95% CI: 1.19–2.92) and OR: 3.25 (95% CI: 1.11–9.54), respectively (Table 3). After stratifying by gender and adjusting for age and BMI, a stronger protective effect was found in males for SNP rs73164856 in OA (OR: 0.17, 95% CI: 0.04–0.73). In females, SNP rs545654 was found to increase the risk of OA (OR: 2.07, 95% CI: 1.15–3.73) (Table 4). To further confirm the relationship of the rs73164856 and rs545654 to the grade of KL determined by gender, we conducted stratification of gender to elucidate the genetic effect. Our results showed that there were two of them with significant differences between OA and the control groups: the rs73164856 in the aldose reductase (AR) gene enhancer was identified to be associated with OA in males, and those with T allele carriers had a protective effect of OA compared to those with the CC genotype (OR: 0.55, 95% CI: 0.33–0.92) (Table S3), especially in patients with severe OA (OR: 0.17, 95% CI: 0.04–0.73). The rs545654 in the neuronal NOS (nNOS) gene was identified to be associated with OA in females, especially in patients with severe OA. T allele carriers had a risk effect of OA compared to those with the CC genotype (OR: 2.07, 95% CI: 1.15–3.73) (Table S4).

### 2.5. Messenger RNA Expression of Putative Genes Among Genotypes of SNPs

This case-control study showed that rs73164856 and rs545654 are associated with the risk of OA. In this study, the GTEx database was used for an expression quantitative trait loci analysis of the messenger RNA (mRNA) expression of SNP loci and downstream genes. We observed that the rs73164856 T allele decreased the expression of the AKR1B15 gene (*p* = 0.00019; Figure S3A). The rs545654 T allele gene increased the expression of the nNOS gene (*p* = 1.2 × 10^−17^; Figure S3B).

## 3. Discussion

In summary, RMCGA demonstrated that rs73164856 and rs545654 play important roles in males and females, respectively, with susceptibility to progressive knee OA, modulating the regulation of critical osteoarthritis-related and gender-oriented genes, *AR*, and *nNOS*. The rs73164856 variant is a strong protective variant factor in males with severe knee OA; rs545654 is a risk factor in females with severe knee OA.

In OA, the activation of proinflammatory cytokines like TNF- or IL-1 by extracellular matrix (ECM) degradation products activates the NF-κB signaling pathway [14,20]. This study identified the upstream regulator, NF-κB, through the case-control RNA-seq literature and then compared the whole genome, NGS SNP data from the TWB database, and ChIP-Seq datasets from the GEO database. Nine SNPs specific to Taiwan were the candidate. However, none of these nine SNPs were associated with OA in the past literature. Only SNP rs2257609 was investigated for its relevance in early-stage non-small-cell lung cancer [21]. A previous study in 2014 also used publicly available genomic data and bioinformatics platforms to provide additional evidence for the TFBSs of SNPs of the estrogen receptor-α (ERα)-regulating sequence at 21q22.3, which are important in determining breast cancer progression [22].

Studies have suggested that gender difference plays a vital role in OA [23]. Women are known to be prone to the development of OA. The factors that might contribute to their susceptibility include thinner cartilage, joint instability, a decline of sex hormone levels, and molecular aspects. Previous studies demonstrate that women have thinner cartilage and are more subject to inflammatory responses, while men may exhibit more stable chondrocyte activity and anabolic compensatory mechanisms [24,25,26,27,28], probably due to TSIX transcript and XIST antisense RNA (TSIX) expression and higher involvement of extracellular matrix turnover compared to those of males [29,30,31]. Transcription factors such as FOXO-mediated transcription factors are generally escalating in females’ osteoarthritic joints, while male cartilage has a lower expression of IGFAL, which is responsible for protein binding IGFs and accelerates its half-life [32].

NF-κB SNPs rs73164856 is located upstream of the AR gene, a key rate-limiting enzyme in the polyol pathway. The expression of AR gene seems susceptible to females in diabetes patients [33], probably due to the loss of ERα [34]. In addition, in a hyperglycemic environment, when hexokinase is saturated and AR is activated, it induces the conversion of glucose to sorbitol. Due to its high polarity, sorbitol itself cannot easily pass through the cell membrane and accumulates in the cell, causing a decrease in Na^+^-K^+^-ATPase activity in the cell, resulting in the loss of inositol and impairment of cellular metabolism and function [35]. The polarization of M1 macrophages can regulate inflammatory responses and tissue damage. Based on a study by Cheng et al., 2021, using bone marrow macrophages from AR KO mice, the absence of the AR gene resulted in the suppression of M1 macrophage polarization and reduced the proinflammatory cytokine activity of macrophages by degrading the IκB kinase (IKK) complex [36]. It has also been suggested that AR stimulates the expression of the inducible nitric oxide synthase (iNOS) gene through lipopolysaccharide (LPS), which increases the concentration of nitric oxide (NO) in various cells, including macrophages. Moreover, the interaction of NO with proteins and nucleic acids will cause the apoptosis of macrophages [37,38].

NF-κB signaling is extensively involved in OA pathology through a variety of patterns. NF-κB signaling induces the secretion of degrading enzymes, such as matrix metalloproteinases (MMP) leading to the degradation of articular cartilage. NF-κB can enhance joint injury by NOS (nitric oxide synthase) and NO (nitric oxide), thereby promoting tissue inflammation, the synthesis of catabolic factors, and the apoptosis of articular chondrocytes [39].

NF-κB SNPs rs545654 is located in the nNOS gene. NO has a wide range of physiological activities, including regulation of vascular tone and neurotransmission. L-arginine (L-Arg) binds to NOS to produce NO. NOS includes nNOS (nNOS, NOS1), immunologic NOS (iNOS, NOS2), and endothelial NOS (eNOS, NOS3) [40,41,42]. Baig et al., 2015, confirmed that nNOS is critical in the inflammatory response by promoting NF-κB activity. NO production by nNOS in macrophages leads to the production of proteolysis by suppressor of cytokine signaling 1 (SOCS1), which increases NF-κB transcriptional activity [43]. In addition, nNOS is also subjective to regulation by estrogen, probably leading to gender difference [44]; a case in point is limb girdle muscular dystrophy [45]. In OA, sex-related differences in immune responses may contribute to the differential expression of nNOS and subsequent NO production. Previous studies have reported that female OA patients exhibit elevated levels of pro-inflammatory cytokines, such as IL-1β, IL-6, and TNF-α. In synovial fluid compared to their male counterparts, potentially reflecting greater sensitivity to inflammatory stimuli in females. [46]. These differences may influence the expression of nNOS and the production of NO, thereby contributing to sex-specific variation in OA progression [47].

The search of these two SNPs in the RegulomeDB database showed that the rs73164856 and rs545654 feature a TF binding site or are DNase sensitive; that is, the chromosome structure may be loose and can be bound by TF, which mean they may be functionally regulated. The GTEx database showed that the extent of abundance AR genes and nNOS rely on different genotypes. However, more research is needed to validate.

Although our RMCGM provides a novel candidate strategy for OA, recent studies have demonstrated that polygenic risk scores (PRS) are increasingly being used to predict OA risk [48,49,50]. These studies utilize publicly available GWAS datasets to calculate individual OA risk scores based on specific SNPs. Furthermore, research by Lacaze et al. and Gill et al. has highlighted emerging biomarkers—such as genomic heterozygosity and age-related genomic risk—as important factors in the development of OA [51,52]. These findings underscore the complex interplay between genetic diversity, aging, and disease progression and emphasize the need for further stratified genomic investigations. Therefore, future integration of PRS approaches with RMCGA-derived strategies may enhance precision in early risk assessment and enable more tailored preventive interventions.

Certain potential limitations of this study might have influenced the results. The differential gene data were obtained from the cartilage of Caucasian patients, which may differ from the RNA-seq results of the Taiwan population; this may have influenced the candidate NF-κB results. Furthermore, the modified NF-κB motif based on Watson–Crick DNA structure rules may be arbitrary, though sequential procedures prove feasible. In addition, TFs do not necessarily bind only to known motifs. More experiments, such as NF-κB ChIP assay, may be needed to validate whether genetic variants impair the binding of NF-κB to affect RNA expression. Sample size limitations resulted in an inability to examine the effects of genes with a MAF of <5% and structural mutations (insertions and deletions) on OA. Although Bonferroni correction was not conducted in an association test, we additionally used a functional test to improve the evidence level by GTEx, which resulted in less type 1 errors compared to the other genetic association studies with Bonferroni correction without a functional test. Therefore, we recommend increasing the sample size in the future to examine SNPs that were not covered in this study in order to obtain more OA-related SNP results.

## 4. Materials and Methods

### 4.1. Study Participants

A case-control study design was adopted with 533 knee OA patients [Kellgren–Lawrence (KL) grade ≥ 2] (134 severe knee OA patients, KL grade ≥ 3) and with 614 healthy individuals (KL grade < 2) at Tri-Service General Hospital, Taiwan, from 2016 to 2021. The sample size was calculated using OpenEpi version 3.0 and came out to be 932 [53]. All participants’ demographic and clinical characteristics were gathered using questionnaires and medical records.

### 4.2. Bioinformatic Analysis in Gene Screening

#### Study Flowchart

**Figure 1:**
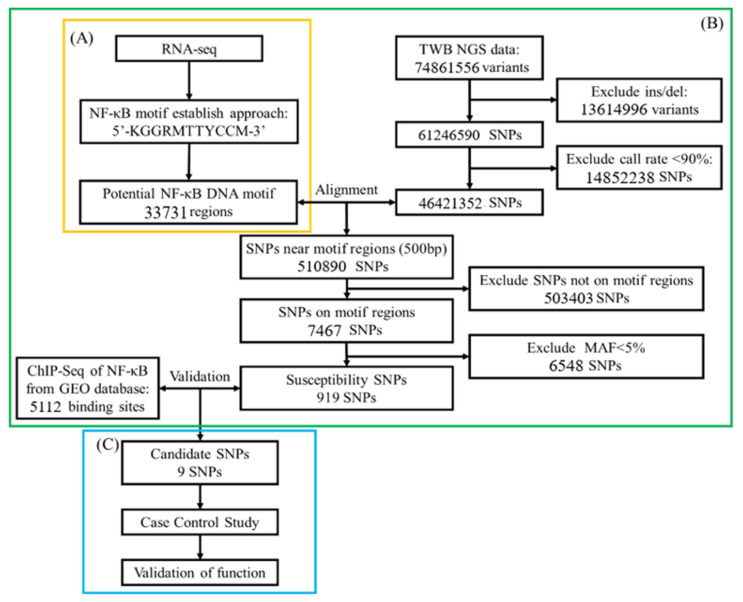
A bioinformatics analysis in the screening of gene processes. (**A**) The upstream TF, NF-κB, associated with OA in the RNA-seq literature [53], searched for its recognition sequence binding position weight matrices (PWMs) using the JASPAR database, and the cut-off points for the occurrence of adenine (A), thymine (T), cytosine (C), and guanine (G) were set as the threshold values. To identify potential binding sites, the human gene sequence data GRCh37-hg19 were downloaded and compared with the candidate TF recognition sequence (motif). (**B**) The NGS data (74,861,556 variants), insertion and deletion, and call rate < 90% were excluded from the TWB database, and the retained loci (46,421,352 variants) were compared with the potential binding sites of NF-κB to identify potential binding sites. The potential binding sites were then compared with the NF-κB ChIP-seq data from the GEO database to obtain the candidate SNPs. (**C**) The candidate SNPs were analyzed for correlation with OA in a case-control study, and their functionality was verified using the GTEx database.

One of the upstream regulators, herein NF-κB, associated with OA was derived from differentially expressed genes (DEGs) in RNA-seq analysis [19]. We used position weight matrices of NF-κB motif from the JASPAR database (https://jaspar.genereg.net/ (accessed on 1 February 2022)) [54] and modified this motif according to Watson–Crick DNA structure rules, with A or T = 30% and C or G = 20% as the threshold value [18]. To select putative genome-wide NF-κB binding sites, we used an established NF-κB motif to align the NCBI database (Figure 1A).

Simultaneously, we used the NGS data of 1517 people; the data were available from the TWB database and included 74,861,556 genetic variants. We excluded variants with insertions and deletions (13,614,996) and a call rate <90% (14,852,238 SNPs) in TWB. An intersection of putative NF-κB binding sites and 46,421,352 SNPs was computed. Those SNPs with 500 bp across NF-κB binding sites upstream and downstream were kept; next, those SNPs with a minor allele frequence <5% were excluded. Susceptible SNPs were mapped to the NF-κB ChIP-seq dataset (GSE55105) to serve as candidate SNPs (Figure 1B).

Participants’ DNA from cases and controls was sequenced to validate those candidate SNPs. Functional assessment was confirmed using the GTEx-portal database (https://www.gtexportal.org (accessed on 1 February 2022)) [55] (Figure 1C).

### 4.3. Genomic DNA Extraction and SNP Genotyping

Genomic DNA was isolated from the peripheral blood samples using the standard procedures for proteinase K (Invitrogen Corp., Carlsbad, CA, USA) digestion and the phenol/chloroform method. Nine SNPs, mentioned above, in the TFBSs were genotyped by iPLEX Gold SNP genotyping [56].

### 4.4. Ethics

This study was reviewed and approved by the institutional ethics committee of TSGH (2-102-05-028). After a detailed explanation of the study objectives, written informed consent was obtained from all participants. All clinical and biological samples were collected and DNA was genotyped after obtaining patient consent.

### 4.5. Statistical Analysis

Continuous variables were reported as the mean ± SD and were tested using *t*-tests. Genotype and allelic frequencies were compared between patients with OA and healthy individuals using chi-squared or Fisher’s exact test. Logistic regression analysis was performed to estimate OR and 95% CI with adjustment for age and gender [10]. The analysis was performed using allele type, genotype, dominant, and recessive models. Statistical analyses were performed using SPSS 22.0 (SPSS Inc., Chicago, IL, USA) and R 3.5.1 (R Project for Statistical Computing, Vienna, Austria). A *p*-value of <0.05 was considered statistically significant.

## 5. Conclusions

This study highlights sex-specific SNPs within NF-κB binding sites that are associated with severe osteoarthritis and confirms the utility of the RMCGA framework. Future integration of PRS approaches with RMCGA-derived strategies may enhance precision in early risk assessments and enable more tailored preventive interventions.

## Figures and Tables

**Table 1:** Characteristics of OA patients and control subjects.

Independent Variables	Control (n = 614)	OA (n = 533)	*p*-Value	Severe OA(n = 134)	*p*-Value
Gender			0.015 *		0.014 *
Male	268 (43.6%)	195 (36.6%)		43 (32.1%)	
Female	346 (56.4%)	338 (63.4%)		91 (67.9%)	
Age	72.48 ± 6.80	73.31 ± 6.94	0.043 *	75.85 ± 7.08	<0.001 *
BMI	23.94 ± 3.41	24.38 ± 3.48	0.042 *	25.19 ± 3.20	<0.001 *

OA: Kellgren-Lawrence (KL) grade ≥ 2; Severe OA: Kellgren–Lawrence (KL) grade ≥ 3. *: *p* < 0.05.

**Table 2:** Genotype distribution of NF-κB binding sites SNPs with OA cases and control group.

SNPs	Control	OA	HWE	Crude-OR (95% CI)	Adj-OR (95% CI)
rs11826681 C/G			0.961		
CC	217 (36.0%)	206 (38.9%)		1.00	1.00
CG	300 (49.8%)	241 (45.6%)		0.85 (0.66–1.09)	0.85 (0.65–1.12)
GG	85 (14.1%)	82 (15.5%)		1.02 (0.71–1.45)	0.98 (0.67–1.43)
rs2257609 G/A			0.630		
GG	205 (33.9%)	193 (36.8%)		1.00	1.00
GA	294 (48.6%)	238 (45.3%)		0.86 (0.66–1.12)	0.82 (0.63–1.08)
AA	106 (17.5%)	94 (17.9%)		0.94 (0.67–1.32)	0.90 (0.63–1.28)
rs3749606 C/T			0.957		
CC	431 (71.4%)	372 (71.3%)		1.00	1.00
CT	163 (27.0%)	141 (27.0%)		1.00 (0.77–1.31)	1.03 (0.78–1.37)
TT	10 (1.7%)	9 (1.7%)		1.04 (0.42–2.59)	0.96 (0.37–2.53)
rs4702701 A/G			0.911		
AA	528 (87.0%)	462 (87.5%)		1.00	1.00
AG	77 (12.7%)	64 (12.1%)		0.95 (0.67–1.35)	0.90 (0.62–1.31)
GG	2 (0.3%)	2 (0.4%)		1.14 (0.16–8.15)	1.06 (0.15–7.63)
rs545654 C/T			0.951		
CC	283 (46.6%)	238 (45.2%)		1.00	1.00
CT	260 (42.8%)	238 (45.2%)		1.09 (0.85–1.39)	1.10 (0.85–1.43)
TT	64 (10.5%)	50 (9.5%)		0.93 (0.62–1.40)	0.94 (0.62–1.43)
rs7256865 T/G			0.298		
TT	451 (74.5%)	396 (75.0%)		1.00	1.00
TG	137 (22.6%)	121 (22.9%)		1.01 (0.76–1.33)	1.03 (0.77–1.39)
GG	17 (2.8%)	11 (2.1%)		0.74 (0.34–1.59)	0.77 (0.35–1.68)
rs73164856 C/T			1.000		
CC	453 (75.1%)	428 (81.8%)		1.00	1.00
CT	141 (23.4%)	89 (17.0%)		0.67 (0.50–0.90) *	0.68 (0.50–0.93) *
TT	9 (1.5%)	6 (1.1%)		0.71 (0.25–2.00)	0.52 (0.17–1.57)
rs77836284 C/T			0.986		
CC	504 (83.2%)	450 (85.9%)		1.00	1.00
CT	98 (16.2%)	71 (13.5%)		0.81 (0.58–1.13)	0.75 (0.53–1.07)
TT	4 (0.7%)	3 (0.6%)		0.84 (0.19–3.77)	0.81 (0.18–3.69)
rs79975923 A/T			0.720		
AA	420 (69.5%)	365 (69.3%)		1.00	1.00
AT	170 (28.1%)	149 (28.3%)		1.01 (0.78–1.31)	1.02 (0.78–1.35)
TT	14 (2.3%)	13 (2.5%)		1.07 (0.50–2.30)	1.31 (0.59–2.94)

Adj-OR: Adjustment with gender, age and BMI; OA: Kellgren–Lawrence (KL) grade ≥ 2; HWE: Hardy–Weinberg equilibrium. *: *p* < 0.05.

**Table 3:** Genotype distribution of NF-κB binding site SNPs with severe OA cases and control group.

SNPs	Control	Severe OA	Crude-OR (95% CI)	Adj-OR (95% CI)
rs11826681 C/G				
CC	217 (36.0%)	52 (39.7%)	1.00	1.00
CG	300 (49.8%)	65 (49.6%)	0.90 (0.60–1.35)	0.90 (0.58–1.41)
GG	85 (14.1%)	14 (10.7%)	0.69 (0.36–1.31)	0.79 (0.41–1.56)
rs2257609 G/A				
GG	205 (33.9%)	44 (33.8%)	1.00	1.00
GA	294 (48.6%)	65 (50.0%)	1.03 (0.68–1.57)	0.91 (0.58–1.45)
AA	106 (17.5%)	21 (16.2%)	0.92 (0.52–1.63)	0.88 (0.48–1.61)
rs3749606 C/T				
CC	431 (71.4%)	88 (67.7%)	1.00	1.00
CT	163 (27.0%)	41 (31.5%)	1.23 (0.82–1.86)	1.35 (0.87–2.10)
TT	10 (1.7%)	1 (0.8%)	0.49 (0.06–3.87)	0.57 (0.07–4.66)
rs4702701 A/G				
AA	528 (87.0%)	111 (84.1%)	1.00	1.00
AG	77 (12.7%)	20 (15.2%)	1.24 (0.73–2.10)	1.11 (0.60–2.02)
GG	2 (0.3%)	1 (0.8%)	2.38 (0.21–26.46)	1.84 (0.16–21.36)
rs545654 C/T				
CC	283 (46.6%)	50 (38.5%)	1.00	1.00
CT	260 (42.8%)	66 (50.8%)	1.44 (0.96–2.15)	1.86 (1.19–2.92) *
TT	64 (10.5%)	14 (10.8%)	1.24 (0.65–2.38)	1.48 (0.75–2.93)
rs7256865 T/G				
TT	451 (74.5%)	97 (73.5%)	1.00	1.00
TG	137 (22.6%)	33 (25.0%)	1.12 (0.72–1.74)	1.11 (0.69–1.79)
GG	17 (2.8%)	2 (1.5%)	0.55 (0.12–2.41)	0.63 (0.14–2.88)
rs73164856 C/T				
CC	453 (75.1%)	107 (81.7%)	1.00	1.00
CT	141 (23.4%)	22 (16.8%)	0.66 (0.40–1.08)	0.72 (0.43–1.23)
TT	9 (1.5%)	2 (1.5%)	0.94 (0.20–4.42)	1.05 (0.22–5.02)
rs77836284 C/T				
CC	504 (83.2%)	113 (85.6%)	1.00	1.00
CT	98 (16.2%)	19 (14.4%)	0.86 (0.51–1.47)	0.80 (0.44–1.46)
TT	4 (0.7%)	0 (0.0%)	0.00 (0.00–Inf)	0.00 (0.00–Inf)
rs79975923 A/T				
AA	420 (69.5%)	84 (65.6%)	1.00	1.00
AT	170 (28.1%)	38 (29.7%)	1.12 (0.73–1.71)	1.10 (0.69–1.74)
TT	14 (2.3%)	6 (4.7%)	2.14 (0.80–5.74)	3.25 (1.11–9.54) *

Adj-OR: Adjustment with gender, age, and BMI; Severe OA: Kellgren–Lawrence (KL) grade ≥ 3. *: *p* < 0.05.

**Table 4:** Genotype distribution of NF-κB binding site SNPs with severe OA stratified by gender.

SNPs	Male		Female	
	Crude-OR (95% CI)	Adj-OR (95% CI)	Crude-OR (95% CI)	Adj-OR (95% CI)
rs11826681 C/G				
CC	1.00	1.00	1.00	1.00
CG	0.65 (0.33–1.29)	0.62 (0.30–1.27)	1.06 (0.63–1.76)	1.15 (0.64–2.05)
GG	0.50 (0.16–1.57)	0.57 (0.18–1.83)	0.80 (0.36–1.76)	1.04 (0.45–2.42)
rs2257609 G/A				
GG	1.00	1.00	1.00	1.00
GA	1.12 (0.56–2.23)	0.95 (0.46–1.98)	0.96 (0.56–1.64)	0.91 (0.50–1.65)
AA	0.50 (0.16–1.58)	0.44 (0.14–1.44)	1.14 (0.58–2.26)	1.17 (0.56–2.47)
rs3749606 C/T				
CC	1.00	1.00	1.00	1.00
CT	0.89 (0.42–1.91)	0.97 (0.44–2.13)	1.38 (0.84–2.27)	1.58 (0.91–2.74)
TT	0.00 (0.00–Inf)	0.00 (0.00–Inf)	0.54 (0.07–4.40)	0.83 (0.10–7.29)
rs4702701 A/G				
AA	1.00	1.00	1.00	1.00
AG	0.93 (0.37–2.36)	0.99 (0.38–2.58)	1.53 (0.79–2.98)	1.22 (0.56–2.69)
GG	6.22 (0.38–101.72)	3.82 (0.23–63.77)	0.00 (0.00–Inf)	0.00 (0.00–Inf)
rs545654 C/T				
CC	1.00	1.00	1.00	1.00
CT	1.55 (0.78–3.07)	1.60 (0.78–3.26)	1.40 (0.84–2.32)	2.07 (1.15–3.73) *
TT	0.78 (0.21–2.84)	0.70 (0.18–2.64)	1.51 (0.70–3.26)	2.24 (0.98–5.12)
rs7256865 T/G				
TT	1.00	1.00	1.00	1.00
TG	0.96 (0.45–2.06)	1.07 (0.48–2.35)	1.22 (0.71–2.10)	1.11 (0.61–2.04)
GG	0.00 (0.00–Inf)	0.00 (0.00–Inf)	0.90 (0.19–4.25)	0.97 (0.19–4.95)
rs73164856 C/T				
CC	1.00	1.00	1.00	1.00
CT	0.16 (0.04–0.67) *	0.17 (0.04–0.73) *	0.97 (0.55–1.70)	1.22 (0.66–2.25)
TT	0.00 (0.00–Inf)	0.00 (0.00–Inf)	1.10 (0.22–5.40)	1.75 (0.34–9.02)
rs77836284 C/T				
CC	1.00	1.00	1.00	1.00
CT	0.90 (0.35–2.26)	0.99 (0.38–2.58)	0.83 (0.43–1.60)	0.81 (0.37–1.75)
TT	0.00 (0.00–Inf)	0.00 (0.00–Inf)	0.00 (0.00–Inf)	0.00 (0.00–Inf)
rs79975923 A/T				
AA	1.00	1.00	1.00	1.00
AT	0.85 (0.39–1.84)	0.81 (0.36–1.82)	1.29 (0.77–2.15)	1.38 (0.78–2.47)
TT	8.38 (2.12–33.16) *	11.96 (2.48–57.64) *	0.42 (0.05–3.34)	0.80 (0.10–6.76)

Adj-OR: Adjustment with age and BMI; Severe OA: Kellgren–Lawrence (KL) grade ≥ 3. *: *p* < 0.05.

## Data Availability

The data presented in this study are available on request from the corresponding author. The data are not publicly available due to privacy issues.

## Supplementary Materials

The following supporting information can be downloaded at: https://www.mdpi.com/article/10.3390/ijms26094117/s1.
