# Supplementary material for: Dissect Gender-Dependent Susceptibility SNPs in Progressive Osteoarthritis Using Regulator Motif Candidate of Genetic Association Strategy (RMCGA)
Source: Int J Mol Sci. 2025 Apr 26;26(9):4117. doi: 10.3390/ijms26094117 (PMC12071535; doi:10.3390/ijms26094117)
Supplement: Supplementary file 1 [file ijms-26-04117-s001.zip › ijms-3576617-supplementary.pdf]

Supplemental Materials

Figure S1. Demonstration of potential NF-κB binding motif

Figure S2. Potential numbers of NF-κB binding sites across chromosomes

Figure S3. The eQTL violin plot of (A) rs73164856 and (B) rs545654

Table S1. Candidates SNPs on NF-κB binding sites

Table S2. Genotype distribution of NF-κB binding sites SNPs with OA stratified by gender

Table S3. The allele frequency of rs73164856 for the NF-κB binding sites SNPs with OA

malegroup

Table S4. The allele frequency of rs545654 for the NF-κB binding sites SNPs with OA female group

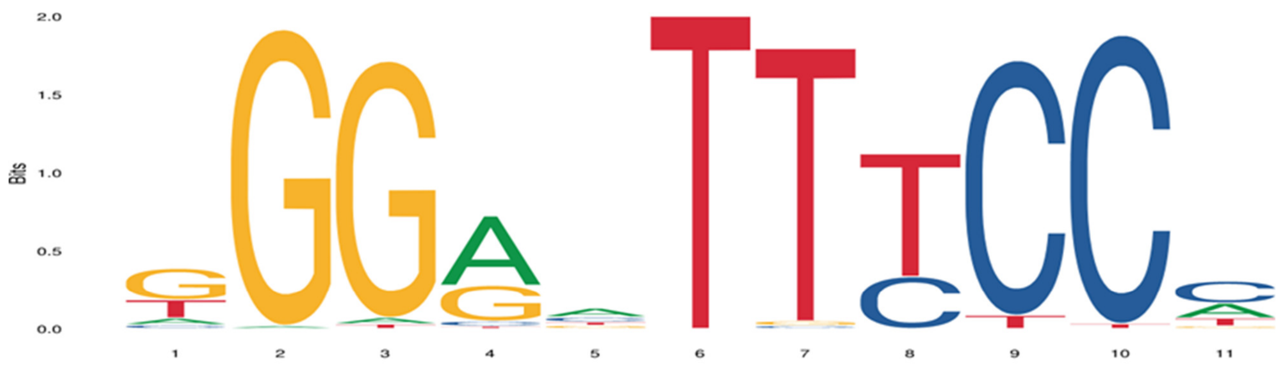

A	[0.27	0.25	0.00	0.00	1.00	0.03	0.00	0.00	1.00	0.01	0.14	]
C	[0.02	0.35	0.00	0.00	0.00	0.48	0.00	1.00	0.00	0.20	0.28	]
G	[0.33	0.37	0.00	0.92	0.00	0.38	0.00	0.00	0.00	0.05	0.27	]
T	[0.38	0.03	1.00	0.08	0.00	0.11	1.00	0.00	0.00	0.74	0.31	]

Figure S1. Demonstration of potential NF-κB binding motif

The recognition sequence is 5’-KGGRMTTYCCM-3’.

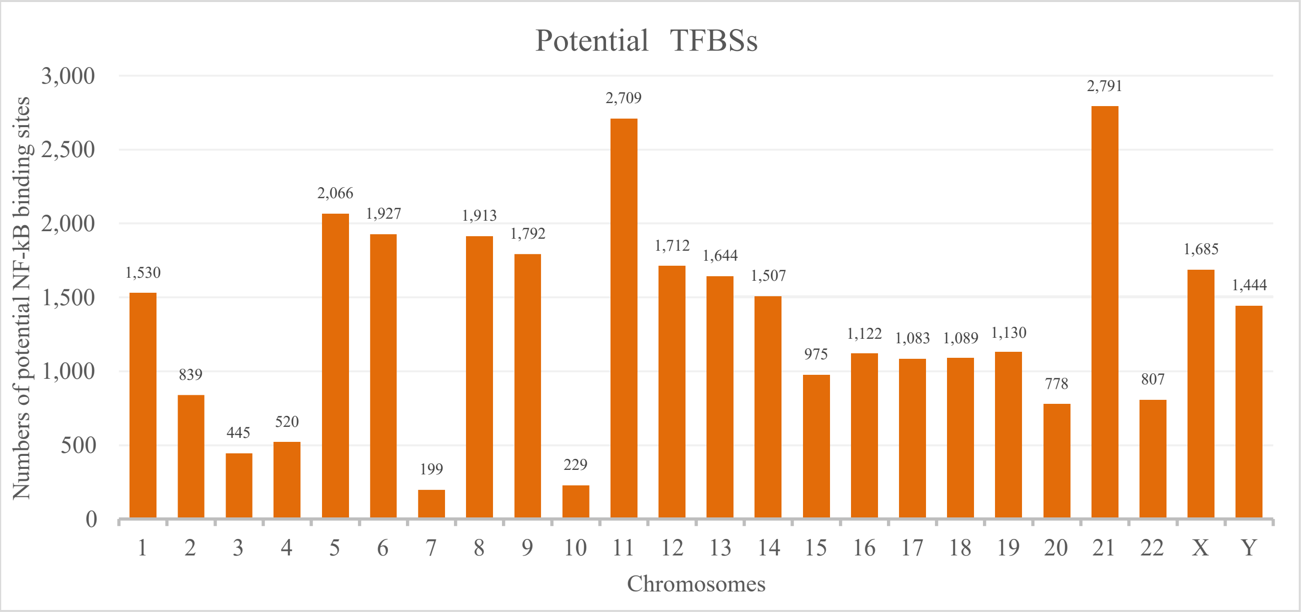

Figure S2. Potential numbers of NF-κB binding sites across chromosomes

The human gene sequence of GRCh37-hg19 was downloaded from the NCBI website, and the NF-κB candidate sequence 5'-KGGRMTTYCCM-3' was used for comparison. 33,731 potential binding sites were identified.

(A) 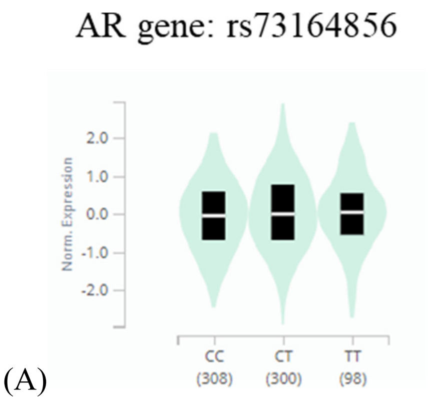

(B) 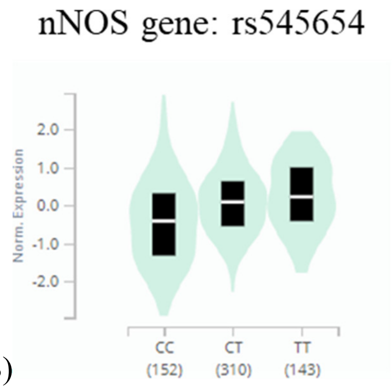

Figure S3. The eQTL violin plot of (A) rs73164856 and (B) rs545654

(A) NF-κB binding site rs7316485 is located at the AR gene position on chromosome 7, and T allele decreases AKR1B15 gene expression in skeletal muscle tissues (p = 0.00019).

(B) NF-κB binding site rs545654 is located at the nNOS gene position on chromosome 12, and the T allele elevates nNOS gene expression in lower limb skin tissues (p = 1.2e-17).

Table S1. Candidates SNPs on NF-κB binding sites

Chromosome: Location	SNPs	Call rate	MAF	DNA sequence near the SNP	Putative Genes
chr11:30323336	rs11826681	94.5%	40.1%	ACGTGAGGGG[C/T]ATTTCCAGCT	ARL14EP
chr17:18961868	rs2257609	98.2%	39.0%	CTCTGAGGGG[G/A]TTCCCAGAGT	SLC5A10
chr5:91102100	rs3749606	100%	15.3%	ATGGGATTTC[C/T]AAGGTTGTTC	ADGRV1
chr5:10578191	rs4702701	100%	12.9%	GGGACTTTCC[A/G]GTTTTTTGTT	ANKRD33B
chr12:117339244	rs545654	99.9%	31.8%	TCCTCAGCCA[C/T]GGAATTCCCC	nNOS
chr19:45515149	rs7256865	96.2%	13.1%	GCCTAGGGAC[T/G]TTCCCTGCCC	VASP
chr7:134549099	rs73164856	99.9%	10.1%	TGACTCATTC[C/T]GGGCTTCCCC	AKR1B15
chr16:86520230	rs77836284	99.9%	9.0%	TGGGGATTTC[C/T]CGCTCGGCTG	Non-determined
chr8:127552997	rs79975923	99.3%	16.0%	AGAGCATGGA[A/T]TTCCCCAAAG	Non-determined

Note. Determination of putative genes derived from NCBI.

Table S2. Genotype distribution of NF-κB binding sites SNPs with OA stratified by gender

SNPs	Male		Female	
	Crude-OR (95% CI)	Adj-OR (95% CI)	Crude-OR (95% CI)	Adj-OR (95% CI)
rs11826681 C/G				
CC	1.00	1.00	1.00	1.00
CG	1.00 (0.67 - 1.49)	1.01 (0.66 - 1.53)	0.76 (0.54 - 1.06)	0.76 (0.54 - 1.08)
GG	0.86 (0.47 - 1.56)	0.91 (0.49 - 1.69)	1.09 (0.69 - 1.72)	1.03 (0.64 - 1.67)
rs2257609 G/A				
GG	1.00	1.00	1.00	1.00
GA	0.88 (0.58 - 1.33)	0.89 (0.58 - 1.37)	0.83 (0.59 - 1.17)	0.78 (0.55 - 1.12)
AA	0.91 (0.53 - 1.55)	0.88 (0.51 - 1.53)	0.96 (0.61 - 1.49)	0.91 (0.57 - 1.44)
rs3749606 C/T				
CC	1.00	1.00	1.00	1.00
CT	1.23 (0.81 - 1.87)	1.24 (0.80 - 1.93)	0.87 (0.62 - 1.23)	0.92 (0.64 - 1.31)
TT	1.45 (0.20 - 10.41)	1.24 (0.17 - 8.96)	0.87 (0.31 - 2.44)	0.86 (0.28 - 2.63)
rs4702701 A/G				
AA	1.00	1.00	1.00	1.00
AG	0.77 (0.44 - 1.33)	0.75 (0.43 - 1.33)	1.15 (0.71 - 1.84)	1.04 (0.63 - 1.73)
GG	2.67 (0.24 - 29.65)	2.21 (0.20 - 24.74)	0.00 (0.00 - Inf)	0.00 (0.00 - Inf)
rs545654 C/T				
CC	1.00	1.00	1.00	1.00
CT	0.94 (0.64 - 1.40)	0.90 (0.60 - 1.35)	1.19 (0.87 - 1.64)	1.27 (0.90 - 1.77)
TT	0.81 (0.42 - 1.55)	0.77 (0.39 - 1.49)	1.02 (0.60 - 1.73)	1.10 (0.64 - 1.89)
rs7256865 T/G				
TT	1.00	1.00	1.00	1.00
TG	0.98 (0.63 - 1.53)	1.01 (0.64 - 1.61)	1.03 (0.72 - 1.48)	1.05 (0.72 - 1.54)
GG	1.19 (0.42 - 3.36)	1.27 (0.43 - 3.73)	0.45 (0.14 - 1.48)	0.44 (0.13 - 1.46)
rs73164856 C/T				
CC	1.00	1.00	1.00	1.00
CT	0.56 (0.34 - 0.91)*	0.55 (0.33 - 0.92)*	0.73 (0.50 - 1.07)	0.79 (0.53 - 1.16)
TT	1.23 (0.17 - 8.86)	0.52 (0.05 - 5.90)	0.54 (0.16 - 1.88)	0.55 (0.16 - 1.91)
rs77836284 C/T				
CC	1.00	1.00	1.00	1.00
CT	0.95 (0.57 - 1.61)	0.91 (0.52 - 1.58)	0.73 (0.48 - 1.12)	0.68 (0.43 - 1.07)
TT	0.68 (0.06 - 7.59)	0.63 (0.06 - 7.08)	0.98 (0.14 - 7.02)	1.06 (0.14 - 7.68)
rs79975923 A/T				
AA	1.00	1.00	1.00	1.00
AT	0.94 (0.62 - 1.42)	0.96 (0.62 - 1.48)	1.07 (0.76 - 1.50)	1.09 (0.76 - 1.56)
TT	2.74 (0.81 - 9.30)	3.64 (0.94 - 14.14)	0.51 (0.17 - 1.52)	0.64 (0.21 - 1.94)

Note. Adj-OR: Adjustment with age and BMI; OA: Kellgren-Lawrence (KL) grade ≥ 2.

*p<.05.

Table S3. The allele frequency of rs73164856 for the NF-κB binding sites SNPs with OA male group

SNP	Model	Grade	Crude-OR (95% CI)	Adj-OR (95% CI)
rs73164856 C/T	Genotype model			
	CT/CC	G2, G3, and G4	0.56 (0.34 - 0.91)*	0.55 (0.33 - 0.92)*
	CT/CC	G3 and G4	0.16 (0.04 - 0.67)*	0.17 (0.04 - 0.73)*
	TT/CC	G2, G3, and G4	1.23 (0.17 - 8.86)	0.52 (0.05 - 5.90)
	TT/CC	G3 and G4	0.00 (0.00 - Inf)	0.00 (0.00 - Inf)
	Allele model			
	T/C	G2, G3, and G4	0.63 (0.40 - 0.99)*	0.57 (0.35 - 0.93)*
	T/C	G3 and G4	0.16 (0.04 - 0.66)*	0.17 (0.04 - 0.71)*
	Dominant model			
	CT+TT/CC	G2, G3, and G4	0.58 (0.36 - 0.94)*	0.55 (0.33 - 0.91)*
	CT+TT/CC	G3 and G4	0.15 (0.04 - 0.65)*	0.16 (0.04 - 0.70)*
	Recessive model			
	TT/CC+CT	G2, G3, and G4	1.38 (0.19 - 9.88)	0.59 (0.05 - 6.65)
	TT/CC+CT	G3 and G4	0.00 (0.00 - Inf)	0.00 (0.00 - Inf)

Note. Adj-OR: Adjustment with age and BMI; G2: KL grade 2; G3: KL grade 3; G4: grade.

*p<.05

Table S4. The allele frequency of rs545654 for the NF-κB binding sites SNPs with OA female group

SNP	Model	Grade	Crude-OR (95% CI)	Adj-OR (95% CI)
rs545654 C/T	Genotype model			
	CT/CC	G2, G3, and G4	1.19 (0.87 - 1.64)	1.27 (0.90 - 1.77)
	CT/CC	G3 and G4	1.40 (0.84 - 2.32)	2.07 (1.15 - 3.73)*
	TT/CC	G2, G3, and G4	1.02 (0.60 - 1.73)	1.10 (0.64 - 1.89)
	TT/CC	G3 and G4	1.51 (0.70 - 3.26)	2.24 (0.98 - 5.12)
	Allele model			
	T/C	G2, G3, and G4	1.07 (0.85 - 1.35)	1.12 (0.88 - 1.42)
	T/C	G3 and G4	1.28 (0.90 - 1.80)	1.60 (1.10 - 2.35)*
	Dominant model			
	CT+TT/CC	G2, G3, and G4	1.16 (0.86 - 1.57)	1.23 (0.89 - 1.69)
	CT+TT/CC	G3 and G4	1.42 (0.88 - 2.29)	2.11 (1.20 - 3.69)*
	Recessive model			
	TT/CC+CT	G2, G3, and G4	0.94 (0.56 - 1.55)	0.98 (0.58 - 1.64)
	TT/CC+CT	G3 and G4	1.27 (0.62 - 2.61)	1.53 (0.72 - 3.25)

Note. Adj-OR: Adjustment with age and BMI; G2: KL grade 2; G3: KL grade 3; G4: grade.

*p<.05
